# Ethnic differences in psychosis—Lay epidemiology explanations

**DOI:** 10.1111/hex.12901

**Published:** 2019-04-30

**Authors:** Peter Schofield, Maria Kordowicz, Earl Pennycooke, David Armstrong

**Affiliations:** ^1^ School of Population Health & Environmental Sciences King's College London London UK; ^2^ Lambeth & Southwark Mind London UK

**Keywords:** epidemiology, ethnicity, psychosis

## Abstract

**Background:**

Much research attention has been given to the high rates of psychosis diagnosed in the Black community. However, little has been heard about possible reasons for this from Black African and Caribbean mental health service users themselves.

**Aims:**

To determine how Black African and Caribbean service users perceive and explain these apparent differences.

**Methods:**

We conducted four focus groups between 2014 and 2015 with 35 participants from the Black African and Black Caribbean community in Lambeth and Southwark, South East London, diagnosed with a psychotic illness. Recruitment was through a local voluntary sector organization and other community contacts.

**Results:**

Each group described an elevated risk of psychosis in their community and explanations followed the following themes, with increased rates due to: (a) an accumulation of stressors due to disadvantaged ethnic minority status, (b) further disadvantage due to inequitable experiences of mental health services, (c) an absence of community support and (d) a double stigma: as a result of external discrimination, due to ethnicity, and internal stigma about mental illness from within the Black community itself.

**Conclusions:**

Black mental health service users attributed an elevated risk of psychosis in their community to an accumulation of stressors directly related to ethnic minority status.

## 1 INTRODUCTION

The relationship between ethnicity and psychosis has been a continuing theme in psychiatric epidemiology, for over 80 years since Ødegaard first reported elevated rates of schizophrenia among Norwegian migrants to the USA.[1] There is now a large body of research evidence showing that members of some migrant and ethnic minority groups, and particularly those from Black African and Black Caribbean communities, are more likely to be diagnosed with a psychotic illness, with at least double the risk according to two recent comprehensive reviews.[2, 3]

In recent years, the number of studies on this theme has increased exponentially, partly as a result of the increasing availability of large datasets of psychiatric records in which ethnicity has been recorded. Research in this area is at a critical point where a number of theories have been proposed, along with some supporting evidence, but a convincing explanation has yet to be established. A range of possible causal factors have been proposed, including disadvantaged socio‐economic status, repeated experiences of discrimination and racism, social isolation and lack of access to social capital. While supporting evidence has been given for each, studies often lack a clear overall theoretical framework to explain why migrant and ethnic minority groups are at an increased risk.[4, 5, 6, 7] It has been argued that for research in this field to now progress we need to look in greater detail at the processes behind these differences and, to this end, research would therefore benefit from qualitative study designs.[8, 9, 10] In this way, phenomena that might otherwise be missed using administrative or survey data could be explored in much greater depth, as could the specific social and geographical contexts in which they occur. We propose that one useful starting point would be the accounts of ethnic minority service users themselves, investigating those explanatory factors they think are most relevant. This would also have important secondary benefits; this is a politically sensitive topic, and the way findings have been disseminated has in the past met with a hostile reception, in part, because authors may have failed to recognize how their work might be interpreted by the Black community.[11, 12]

There is, however, a paucity of explanatory accounts from Black and minority ethnic minority service users. While there are some relevant studies, they have not approached mental health service users directly about their explanations for the increased rates of severe mental illness in their community.[9, 13] We argue that it is important to understand how members of Black and minority ethnic (BME) groups themselves perceive issues of concern to them, not only to inform research, but also to better communicate the results of research and how we translate these into interventions. The value of lay epidemiology has already been demonstrated in the field of cardiovascular disease health promotion, where lay accounts of disease aetiology have helped inform how health promotion initiatives are communicated to patient groups.[14] Furthermore, lay accounts may be tied to clinical outcomes by shedding light on perceptions and attitudes to treatment[15] and narrowing the gulf between professional and service user discourses.[16, 17] Lay epidemiology can also mobilize the expertise that mental health service users develop about their own condition.[18]

We therefore set out to investigate Black mental health service users’ explanatory accounts of ethnic differences in the diagnosis of psychotic illness. We used focus groups as we were interested in hearing how Black mental health service users explained the experience of mental health problems to each other and as a group.[19] Focus groups have already been successfully used to explore lay understandings of stigma[20] and help seeking behaviours in mental health.[21] The study set out to answer two questions: firstly, how do BME mental health service users perceive ethnic differences in psychosis and secondly, what are their explanations for these differences?

## 2 METHODS

### 2.1 Participants and setting

The study was conducted in Lambeth and Southwark (South East London) an area with the second highest concentration, after neighbouring Lewisham, of Black Caribbean and Black African communities in the UK (Census 2011).

Participants were recruited to the study as a purposive sample, on the basis of ethnicity and lived experience of psychotic illness, through MIND in Lambeth and Southwark (a local voluntary sector mental health organization with extensive experience working with BME mental health service users) and other community contacts. Inclusion criteria included: being over 18 years of age, having been diagnosed with a psychotic illness in the past, or currently, and being of Black (African or Caribbean) origin. Participants were contacted through a variety of different routes including posters and leaflets advertising the groups placed in local community resources, approaching potential participants involved in Lambeth and Southwark Mind, and approaching existing participants’ contacts who met the recruitment criteria (snowball sampling).

A series of four groups were held in different community settings in Lambeth and Southwark between July 2014 and July 2015 including a total of 35 participants (69% male/31% female).

### 2.2 Focus group procedure

Each participant was given an information sheet, and the purpose of the study and the implications of taking part were explained to them before obtaining consent to participate. The focus groups were chaired by a BME former mental health service user with extensive experience working with this client group in a therapeutic environment. PS introduced the project and provided an overview of the study aims at the beginning of each group and helped with their facilitation. PS took notes during the discussion, which then helped to facilitate the interpretation and analysis of the focus group transcripts.

Each group lasted between 60 and 90 minutes, and discussions were semi‐structured with discussion topics and prompt questions outlined in the study topic guide. The study topic guide was intentionally broad to avoid framing the discussions according to pre‐defined academic concepts, and a group facilitator was deliberately chosen who did not have an academic background. Also, as the groups progressed, the topic guide evolved to better reflect the priorities of participants. All meetings were audio‐recorded, recorded with participants consent, and transcribed at which point any identifying details were removed.

### 2.3 Data analysis

Data were synthesized through a comprehensive process of thematic analysis, aiming to elicit the salient themes arising from the focus group discussions and subsequent feedback. A hybrid approach of inductive and deductive analysis was used.[22] Transcripts and focus group notes were managed using NVivo. Data were coded through detailed reading by two researchers (MK, PS), in close collaboration with the other members of the research team (DA, EP), using an inductive approach. The research team met regularly to reflect on the data and identify and discuss emerging themes in an iterative process. These themes were cross‐checked across the focus group data and notes. An overarching conceptual framework was refined by the research team to enable the data to be synthesized to examine relationships between themes and develop explanatory accounts for the data until a thematic saturation point was achieved, whereby no new themes emerged.

Member checking of emergent findings was carried out by asking the participants, either by email or phone to comment on the accuracy of the themes. All participants were sent a copy of a brief outline of preliminary themes to comment on. Four participants responded at this stage of the analysis, and their feedback was incorporated in the analysis.

## 3 RESULTS

A range of views were expressed in the groups, and several prominent themes emerged. Some participants felt that mental health issues were an individual matter and did not agree that being in a particular group, because of ethnicity or any other characteristic, was relevant. The majority, however, felt that members of the Black community were more likely to be diagnosed with a psychotic illness and that this reflected a greater underlying risk. There was much discussion about how this in turn was related to wider disadvantages. The overarching themes are presented below along with anonymous illustrative quotes.

### 3.1 Within‐ and between‐group comparisons

The characteristics of each focus group, including the way they were recruited, had a bearing on the study results (see Table 1). The first three groups were recruited in the same way, initially drawing on members of a Black mental health support group and also advertising to day centre attendees at a local MIND branch. We then encouraged those who were interested to invite friends and contacts. Group 1 comprised both Black Caribbean and Black African participants, and there were important differences in the accounts of both ethnic groups, for example around the question of stigma within their respective communities. We therefore decided to run subsequent groups for each ethnic group separately. Focus groups 1 (mixed) and 3 (Black Caribbean) were similar in size, and the tone of the discussion was also similar. Participants were highly engaged with the topic, and there were often heated exchanges of views. For example, many felt strongly that racism and discrimination were central to explanations for increased rates of mental illness. Others, however, felt that views expressed were overly negative and hostile to the majority White population. This was a particular cause of friction for some participants in group 3. Focus group 2 (Black African) was much smaller and more cohesive, with participants tending to reinforce the points others in the group had made. This group were particularly concerned about the role stigma plays in the Black African community and how this can exacerbate the experience of mental distress. They placed less weight on discrimination and racism as contributory factors (compared to groups 1 and 3). Focus group 4 (Black African) were recruited through a local GP practice and were more divided than previous groups, with participants split between those who saw social factors, such as discrimination, as relevant and those who felt that mental health should be primarily an individual responsibility. The latter were therefore more inclined to look for individual explanations rather than examine factors operating at the level of the wider BME community, and therefore saw debates about ethnic differences as largely unhelpful and consequently engaged least with the research question.

**Table 1:** Focus group characteristics

Focus group	Broad ethnic category	Gender	Setting	Total attending
1	Mixed Black African/Caribbean	9M 5F	MIND	14
2	Black African	2M 1F	MIND	3
3	Black Caribbean	11M 7F	MIND	18
4	Black African	2M 4F	Local GP practice	6

Note

Six people attended both groups 1 and 3 giving an overall total of 35.

### 3.2 Theme 1: Accumulation of stressors

Much of the groups’ explanatory framework centred on the accumulation of life stresses resulting from multiple social disadvantage, both directly and indirectly related to ethnicity:

We’re dealing with things like poverty, isolation, a lot of people are coming from other countries, asylum seekers … They have stress where they are, they’ve got stress when they come over here. (Focus Group 1)

The ethnic minority experience is not the same as the native experience. Maybe we’ve had more struggles in life, and especially inner city and stuff like that, and basically the struggles that we have in life could turn us in a different direction. (Focus Group 2)

Some talked specifically about employment, and the challenge of securing work was viewed as a significant contributing factor:

It's a stress related illness for instance if you go to get a job and you get turned down that could be stress related so that could build up eventually. (Focus Group 3)

Participants also expressed their frustration at having encountered discrimination in their search for work:

Those root causes of disillusionment, going for jobs, applying for jobs, you know, and it makes you question why such things still go on ‘cos you’re being told there's nothing like racism. (Focus Group 2)

Yet, it was the *accumulation* of these stressful experiences, typically linked to social deprivation and the consequences of institutional racism, that was highlighted as the major risk factor:

(…) going for jobs and getting knocked down…you get knocked down. You get knocked down when you go for house – you get the worst kind of housing. Sometimes, even when you are going to a shop, you get knocked down by your neighbours. You get knocked down when you go into the post office. When you have this thing happen to you repeatedly, you understand, after a while you do get psychotic, (Focus Group 3)

On a daily basis your self‐esteem, your self‐respect [all agreeing], your way of, you can no longer think positively, you understand, you’re always under stress, flight or fight, yeah … the chemicals for flying are always building up in you, they’re never going down, your pulse rate always going up (Focus Group 3)

This was not just an individual experience but was felt to be something accumulated, and inherited, over generations:

There's something in our psyche that comes down from all these generations where we’re feeling disempowered (Focus Group 1)

Therefore, the impact of everyday problems became heightened:

you have to deal with your own personal issues, so for example whether it's a marriage breakdown, whether it's a pregnant, a divorce, a funeral. Which everyone goes through, but you’re picking up the daily stresses combined with the ones that come from our ancestors. (Focus Group 1)

Often a sense of social adversity was heightened for those not born in the UK due to the unmet expectations they had when they arrived:

Your expectations are high and then you come into this country now and people say there's no racism, there is no injustice, but there is. (Focus Group 2)

For some, the contrast was most acute when compared with expectations of what they could achieve:

You’re swimming against the tide, yeah, you’ve got expectations and other people have got less expectations than you, of course it's going to create, what, situations in which you become psychotic. (Focus Group 3)

Further, the rates of psychosis, expressed in terms of recovery, were contrasted with their country of origin:

People recover in the Caribbean and Africa and they recover in Asia, whereas over here we’re just in a vicious circle of poverty where you’re just going round and round and round. (Focus group 1)

### 3.3 Theme 2: Experience of discrimination within mental health services

While many thought the underlying risk of psychosis was greater in the Black community, it was also a commonly held view that they were more likely to be diagnosed:

People from the Black community are quickly diagnosed as being psychotic. (Focus group 1)

Many viewed this as the result of behaviour being misinterpreted due to their ethnic background:

A lot of us have been misdiagnosed because somebody thinks that if you’re somebody from the Caribbean and you happen to mention something like, you had a vision: ‘So you’re seeing things then’ (Focus group 1)

The groups conveyed some uncomfortable paradoxes, where the frustration of being pigeonholed, seemingly because of their ethnicity, could lead to frustration and anger which then served to reinforce the stigma of the “big, Black and dangerous” stereotype.[23]

there's a stereotype (…) when I first came to this country, of Black people being mad, bad, sad and dangerous, that was overwhelming. (Focus Group 3)

If I walked into a place and I said to somebody in a very calm manner, ‘Listen you’re not doing your job, do it’, you know it gets ignored, so I became aggressive, because it's expected: I’m a Black man; who's aggressive. (Focus Group 1)

They tell us you’re this you’re that and that's not the way we are and then sometimes we become that way (Focus Group 3)

However, some felt this also led to situations where their illness went unrecognized, reflecting that they were more likely to receive treatment when most able to express their frustrations, rather than when experiencing negative symptoms. However, it was during such episodes when they were least articulate that they could be most in need of help:

Docile for me that's when I actually really need the help because all I’m doing is sitting in my room (…) but when you’re aggressive that's when the five police cars come around. (Focus Group 1)

Such preconceptions could therefore lead to under‐diagnosis and a failure to provide services until it was too late:

Certain people seem to think that as a Black person to be considered to need the services of the mental health services, you have got to look a particular way, act a particular way. They turn you away at one stage because you don’t fit that bracket. Dr ## says, `Oh. He looked tidy.’ (Focus Group 1)

Some argued that this was due to a lack of cultural awareness among staff:

They’re culturally insensitive, they are not aware of Caribbean African culture (..) we are very spiritual people who believe that people have visions, yeah, and believe that people have enlightenment or whatever you want to term it. And so it's acceptable in our community (Focus Group 1)

Which needed to be addressed through better training:

A whole lot of it is going to come to educating the, educating the frontline staff, the staff who you come into contact with and make a diagnosis. (Focus Group 1)

There was also a common view that Black people were more likely to stay in the mental health system:

I think it can happen to anyone, but a lot of black people get into the mental health system. I think it's like a revolving door. Revolving door, yeah. You see it's a vicious circle. Once you’re in that system it's as if you’re going to be in it for life. (Focus Group 1)

This was partly understood to be because of the limited treatment being offered:

I know a lot of people and you’ll find that they’re still in and out of hospital, in and out of hospital, in and out of hospital. Maybe they’re not getting the right treatment or care that they need. (Focus Group 2)

Less money being put in treatment for Black people, of course there is, that's for sure. Who in this room has been offered, you know, talking therapy when they needed it at the beginning? (Focus Group 3)

Some talked specifically about medication being prescribed inappropriately, and under duress:

Once we got in the system: heavy injections, very high doses of medication. (Focus Group 3)

However, members of one focus group felt that racial discrimination was not a feature of the care they received:

I don’t think that's true (that people from minority groups receive poorer services). I don’t think, because when it comes to receiving any treatments regardless I have never seen any situation: ‘this is for Black people this is for white people’. The medication is all the same. (Focus Group 4)

### 3.4 Theme 3: Absence of community support

The absence of a community support system was also highlighted as a particular risk factor for mental illness in the Black community. For instance, where previously the extended family might have played an important role this was no longer seen to be the case:

We just don’t have support systems. I mean my parents came here back in the 60s and they left their brothers and sisters behind. We didn’t have the support system. (Focus Group 2)

Indeed, countries of origin were recognized for their stronger sense of community support and contrasted with a perceived lack of social support in the UK:

The biggest factor in this country, why most Black people are diagnosed, it is isolation because where we come from, massive families, with good friends, got relatives, we’ve aunties. But when you come here to this country you are alone in your house. (Focus Group 1)

Whereas here when you’re entangled with the services you suddenly realise how few friends or family you have. (Focus Group 1)

Generally, the absence of community support was regarded as an important part of the problem:

There's a lot of support that's absent and would have made a big difference: people calling around to your house to check things are alright, talking to your kids, talking like family, people not closing the doors on families… you know, having community. (Focus Group 2)

Some pointed to the absence of others from the same ethnic background as a factor:

I had some support from neighbours who were the same ethnic minority as me, but then when they moved away I didn’t have any real support, but it depends on how well you get on with the neighbours as well, you know, not everyone has support. (Focus Group 2)

The groups saw community initiatives as an important solution to the problems of isolation and marginalization they faced as Black service users with a diagnosis of severe mental illness:

We need to build institutions within our communities which represent us. (Focus Group 3)

Me, I need to add that in the community, especially for Black community, the people they have to be taught how to help each other. (Focus Group 4)

### 3.5 Theme 4: Stigma within the Black community

In contrast, there was also the commonly expressed view that Black communities, particularly the African community, could be more prone to stigmatize people with mental health problems:

I think they think it's a stigma. I think African people, I think they find it hard to accept that there's mental health problems in their family. I think they find it hard to accept (Focus group 2)

For some, this was associated with traditional beliefs and practices:

I mean back in Africa, back in the day, the way to treat mental illness is to use a whip or chain you down somewhere. (Focus group 2)

So some people think the medication is to beat you up and then release you [from the `Jinn’ (spirits)]. I told them I don't think that's the right thing to do to someone, yeah? (Focus group 4)

More commonly, the experience was a negative reaction from friends and family members:

My dad came to visit me in ## and he told my sister, ##'s gone to a very bad place, ## Hospital and my friends came visiting me, from the church and they were to see if I was okay and that, but being in hospital it was frowned upon and people thought, like `Oh, he's a bad person’. It's hard to explain, like how people treat you and that when you’re mentally ill. Even your own family can disown you (Focus group 2)

## 4 DISCUSSION

We set out to investigate Black mental health service users’ views about and explanations for the elevated rates of psychosis reported among ethnic minority groups. This was perceived by almost all participants as a problem in their community, and a range of explanations were given. Most often this was attributed to an accumulation of stressors that were specific to members of the Black community. Stresses attributed to socio‐economic deprivation and discrimination were seen to be further compounded by inequitable treatment from mental health services and a tendency to over‐diagnose ethnic minorities with a psychotic illness. The absence of community and support networks was also highlighted while some felt that stigma from within the Black community itself was an important factor.

Many of these accounts closely parallel explanations put forward by the academic community. Accumulated social adversity including unemployment, social isolation and poor education has been shown to contribute to the increased risk of psychosis among ethnic minority groups,[7] and it is the cumulative nature of these stressors that is most often attributed a causal role.[7, 24, 25]

For many, the underlying, or fundamental, cause behind these experiences of adversity is racial discrimination[8, 26, 27] and this is as much a unifying theme in the academic literature as it was in the focus group accounts. The role that mental health services play, in terms of both poorer quality and more coercive treatment for ethnic minorities, is also well documented.[28, 29, 30] Focus group members also stressed the importance of social support, and some expressed the feeling that their community was being eroded and no longer able to provide the support needed for those experiencing mental distress. Recently, there has been a growing academic interest in the social context in which ethnic minorities are situated geographically with studies showing evidence that living in a low ethnic density area (where there are few of one's own ethnic group) is a risk factor for psychosis.[6, 31, 32] Often this is attributed to the absence of social support that might otherwise be protective.[9]

Lastly, it has long been recognized that stigma associated with mental illness can itself play a causal role.[33, 34] However, it is only recently that attention has been paid to internal stigma within some ethnic minority groups.[35, 36, 37] Shefer and others report the results of a series of focus groups with different ethnic groups where they observed that members of the Black African community were most likely to report high levels of stigma from within their own community.[35] This mirrors our study findings where this was also more likely to be a feature of the accounts of Black African participants. The potential existence of “external stigma” for Black service users through forms of racism and discrimination from the majority White community may therefore be compounded by “internal stigma” from sections of the Black community reflecting negative views about mental illness. While a sense of community might help ameliorate some of the external stigma, if this is accompanied by internal stigma, then this double stigma could further amplify underlying psychological distress.

### 4.1 Study strengths and limitations

There is now an extensive literature on patients’ explanatory models, that is the way patients make sense of the illnesses they suffer in contrast to formal medical “scientific” explanations. However, this investigation was more concerned with how patients explained illness at a community or population level. The contrast here is between how an individual patient accounted for their illness and how a group of patients explains the patterning of illness across their community—hence the descriptor of “lay epidemiology” and the use of focus group methods to elicit these population perspectives. Inevitably, some of the focus group discussion did relate to individual experiences but in the main all groups managed to give voice to their collective experience and discuss issues they saw as contributing to the increased rates of psychosis in their community. An important benefit of the study was that each group was facilitated by a member of the Black community with experience of mental health service use, and this may help explain why participants were happy to share their views. A further strength of the study was the use of focus groups to elicit accounts pertinent to the experience of the Black community as a whole rather than concentrating solely on individual experiences. We also made sure that our initial findings were shared with participants for their feedback. However, despite an invitation to comment on the emerging findings, only four focus group members responded. While broadly supportive of our findings, feedback was most often concerned with individual mental health experiences alone. This response itself provides some indication of how focus groups differ and the sorts of responses we may have encountered had we instead used individual interviews. However, a focus group methodology is much less likely to be effective at exploring psychological explanations, such as issues of personal identity and acculturation stress which have also been proposed to explain some of these ethnic differences.[38, 39] For this, a complementary series of one‐to‐one interviews could be more relevant.

We also acknowledge a potential bias where coders, already familiar with the academic literature, might frame lay accounts according to already established themes. However, we made sure to use a second coder, from outside of the field of psychosis research, whose independent analysis arrived at a very similar set of themes to the first coder. At the analysis stage, we also involved respondents themselves, circulating summaries of the main themes and incorporating subsequent comments. It is, however, difficult to make a claim for these groups to be truly representative as participants were self‐selected having been motivated to participate in the first place.

The recruitment strategy used will also have influenced the kind of responses received. Given the well‐known difficulties recruiting mental health service users, especially from BME communities[40, 41] we decided to take a pragmatic approach by recruiting participants through a local voluntary sector organization with strong links with the target population. To test whether links with this one organization affected the views expressed, we also recruited one group (focus group 4) from mental health users attending a GP practice. It is notable that this was the least cohesive of the four groups and also the least likely to engage with the research question.

While we were restricted to English speaking participants only for this study, we were able to achieve a balance of Black Caribbean and Black African participants, broadly representative by age and gender. However, we acknowledge that this is a small exploratory study of specific groups of people at a specific time. Despite this, we argue participants were able to speak on behalf of their community to convey something of the universality of their experience and, in this way, contribute to our understanding of ethnic differences in mental health.

## 5 CONCLUSION

Epidemiological research identifies risk factors for mental illness; many of these factors, such as socio‐economic status or gender or age, that seem causally related to mental illness, might not be recognized by patients themselves. Lay epidemiology attempts to explore how lay people account for the experience of illness in their community. As such, it tends to capture reasons rather than causes but it has the potential to reveal the processes by which mental illness comes about. This qualitative study of lay accounts has offered some support for the epidemiologist's risk factors. Deprivation, here presented in a richer and more nuanced way, is clearly recognized as a significant factor in both determining and maintaining mental illness in the Black community. But what is particularly salient, and often missing from epidemiological accounts, is the experience of stigma internal to the Black community. These Black mental health service users report stigma as operating indirectly in exacerbating their state of deprivation but also directly through the reactions of others to their behaviour. On the one hand, these “others” are the mental health services that for some—though not all—continue to show institutional racism, but on the other hand the Black community itself can be a source of stigma given its understanding of mental illness. These lay epidemiology accounts therefore provide insights into questions of aetiology that may otherwise be missed by more conventional risk factor models.

## CONFLICT OF INTEREST

The authors declare that they have no conflicts of interest.

## ETHICAL APPROVAL

The study received ethical approval from the Biomedical Sciences, Dentistry, Medicine and Natural and Mathematical Sciences Research Ethics Subcommittee (BDM RESC), Kings College London (Reference BDM/14/15‐21). Participants provided written informed consent before taking part in the focus groups.

### DATA AVAILABILITY

The data that support the findings of this study are available on request from the corresponding author. The data are not publicly available due to ethical restrictions.
